# Assessment of Preoperative Informed Consent Practices Among Patients Who Had Undergone Major Abdominal Surgery: A Cross-Sectional Study in Northeast India

**DOI:** 10.7759/cureus.88516

**Published:** 2025-07-22

**Authors:** Prajnaparmita Saha, Sambit Debbarman, Debasis Ray, Gowtham K Gudimetla, Suryadipta Ghosh

**Affiliations:** 1 Pharmacology, Agartala Government Medical College, Agartala, IND; 2 General Surgery, Agartala Government Medical College, Agartala, IND

**Keywords:** informed consent, medical ethics, patient's right, preoperative care, surgical patients

## Abstract

Background

Informed consent is essential in surgical care to uphold patient autonomy through clear communication of diagnosis, treatment options, and risks. This study aimed to assess the practice and quality of preoperative informed consent among patients who had undergone major abdominal surgeries at a tertiary care hospital in Northeast India.

Methods

This hospital-based cross-sectional observational study was conducted over two months, April and May 2025, at Agartala Government Medical College & GBP Hospital. All adult patients (≥18 years) who underwent major abdominal surgeries during the study period were recruited through consecutive sampling. Data were collected using a structured questionnaire through bedside interviews conducted between postoperative days 1 and 3. The questionnaire included items related to patient understanding of their condition, surgical procedure, anesthesia, complications, alternative options, and satisfaction with the consent process.

Results

A total of 200 patients participated in the study, with a mean age of 44.3 (±15.0) years; the majority were female (60.5%, 121/200), from urban areas (58.5%, 117/200), and graduates (63%, 127/200). All participants reported being informed about their diagnosis, surgical procedure, anesthesia, and surgical complications. While 86.5% (173/200) were informed about anesthesia-related complications, this varied significantly with patients’ place of residence and education. Similarly, 85.0% (170/200) were informed about alternative treatment options, which was significantly associated with both place of residence and type of surgery. All patients reported being given adequate time to ask questions. Consent was obtained by consultants in 91.5% (183/200) of cases, significantly associated with address and surgery type. In all instances, consent was signed by both patient and attendant, and properly witnessed. No audio or video recording was used. Overall, 94% (188/200) of participants reported satisfaction with the informed consent process, with satisfaction levels varying significantly by place of residence and educational status.

Conclusion

The study reveals generally good adherence to informed consent practices for major abdominal surgeries at this tertiary care hospital in Northeast India. While the overall satisfaction with the consent process was high, there is room for improvement in ensuring consistent and comprehensive information delivery across all patient demographics.

## Introduction

Informed consent represents a fundamental aspect of ethical medical practice, ensuring that patients are actively involved in decisions regarding their care. It is not simply a formality or a signature on a document, but rather a dynamic process of communication between healthcare providers and patients. Through this process, patients receive essential information regarding the nature of their medical condition, proposed interventions or procedures, associated risks and benefits, as well as available alternatives. This allows them to make decisions that align with their values and preferences [1]. Providing clear and relevant information is a key component of the doctor-patient relationship [2]. With increasing emphasis on patient autonomy and shared decision-making, the responsibility to convey understandable information, particularly before surgical or invasive procedures, has become even more crucial. Informed consent should be conducted in a manner that is comprehensible to the patient, using non-technical language. This ensures that consent is not only informed but also voluntary [1,3,4]. The concept of informed consent typically encompasses three essential elements: preconditions, information, and consent itself. Preconditions refer to the patient's cognitive ability and willingness to participate in decision-making. The information component involves disclosing the patient’s diagnosis, prognosis, proposed treatments, potential complications, and alternative options. Consent, as the final element, signifies the patient’s autonomous decision to proceed, ideally documented after thorough understanding and without coercion [3,5].

Globally, various stakeholders are involved in the informed consent process. These include the patients and their families, healthcare professionals such as doctors and nurses, and institutional or legal entities such as regulatory bodies and medical boards [6]. For surgical interventions in particular, the process often involves discussions around anesthesia options, possible complications, blood transfusion needs, and postoperative expectations. Each of these discussions may be guided by specific consent forms tailored to the nature of the procedure. A successful surgical outcome is not solely dependent on technical expertise but also on the trust established between the patient and the healthcare provider. This trust is cultivated by respecting the patient’s autonomy, even when their choices might pose personal risks [7]. Despite legal and ethical frameworks in place, there remain instances where patients report receiving insufficient information prior to surgery [8].

While informed consent practices have been extensively studied in various contexts, there is a paucity of data from the northeastern region of India. Therefore, the present study aimed to evaluate the preoperative informed consent process for patients who had undergone major elective and emergency abdominal surgeries in the Department of General Surgery at a tertiary care hospital in Northeast India.

## Materials and methods

Study design and setting

This was an observational cross-sectional study conducted in the Department of General Surgery, in collaboration with the Department of Pharmacology, at Agartala Government Medical College and GBP Hospital. The study was carried out over a period of two months, during April and May 2025.

Study population and selection Criteria

The study population comprised patients who underwent major elective or emergency abdominal surgeries in the Department of General Surgery at Agartala Government Medical College and GBP Hospital. Patients aged 18 years or older were included in the study. Those below 18 years of age, those who did not provide informed consent, or those with diagnosed mental health disorders or any condition affecting cognitive judgment were excluded.

Sample size and sampling

During the study period, a total of 225 abdominal surgeries were performed. After applying the inclusion and exclusion criteria, 200 patients were deemed eligible and were included in the study. A consecutive sampling technique was employed to recruit participants.

Data collection

Following approval from the Institutional Ethics Committee, eligible patients were screened postoperatively. Written informed consent was obtained from each participant prior to data collection. Data were gathered using a pretested, semi-structured questionnaire administered through face-to-face interviews conducted by the principal investigator. The questionnaire was adapted from previously published studies with modifications done according to the study’s objectives and feasibility [9,10]. Interviews were conducted at the patient’s bedside between postoperative day 1 and day 3, at the earliest point when the patient was deemed comfortable to participate. To ensure unbiased responses, the interviews were conducted in the absence of the treating healthcare personnel. Patient privacy and confidentiality were strictly maintained throughout the process. Each question was explained in the participant’s own language (Bengali), and responses were recorded only after confirming that the participant had clearly understood the question.

Data analysis

Data were managed using Microsoft Excel (Microsoft Corp., Redmond, WA) and analyzed with IBM SPSS Statistics, version 26 (IBM Corp., Armonk, NY). Categorical variables were summarized using frequencies and percentages, while continuous variables were expressed as mean and standard deviation. There were no missing data. The association between various factors and the informed consent process was assessed using the chi-square test/Fisher’s exact test and a p-value of less than 0.05 was considered statistically significant.

## Results

A total of 200 patients participated in the study, with a mean age of 44.3 (±15.0) years, and the majority fell within the 31-60-year age bracket. The majority were female (60.5%, 121/200), from urban areas (58.5%, 117/200), and graduates (63%, 127/200). Elective surgery was most common among the cases. Detailed baseline characteristics are depicted in Table 1.

**Table 1:** Baseline characteristics of participants (n=200)

Variable	Subgroup	Frequency	Percentage
Age group (in years)	18-30	44	22.0
	31-60	123	61.5
	>60	33	16.5
Gender	Female	121	60.5
	Male	79	39.5
Residence	Rural	83	41.5
	Urban	117	58.5
Educational qualification	Illiterate	21	10.5
	Primary school	6	3.0
	Secondary school	46	23.0
	Graduate	127	63.5
Type of procedure	Elective	158	79.0
	Emergency	42	21.0

The most common surgical condition observed was cholecystitis, accounting for 101 cases. This was followed by hernia (26 cases), appendicitis or appendicular abscess (21 cases), and both common bile duct stones and hollow viscus perforation, each contributing 12 cases. Additionally, there were nine cases of malignancy, six cases each of abdominal trauma (either blunt or perforating) and intestinal obstruction, and two cases of splenic cyst or abscess.

**Figure 1:**
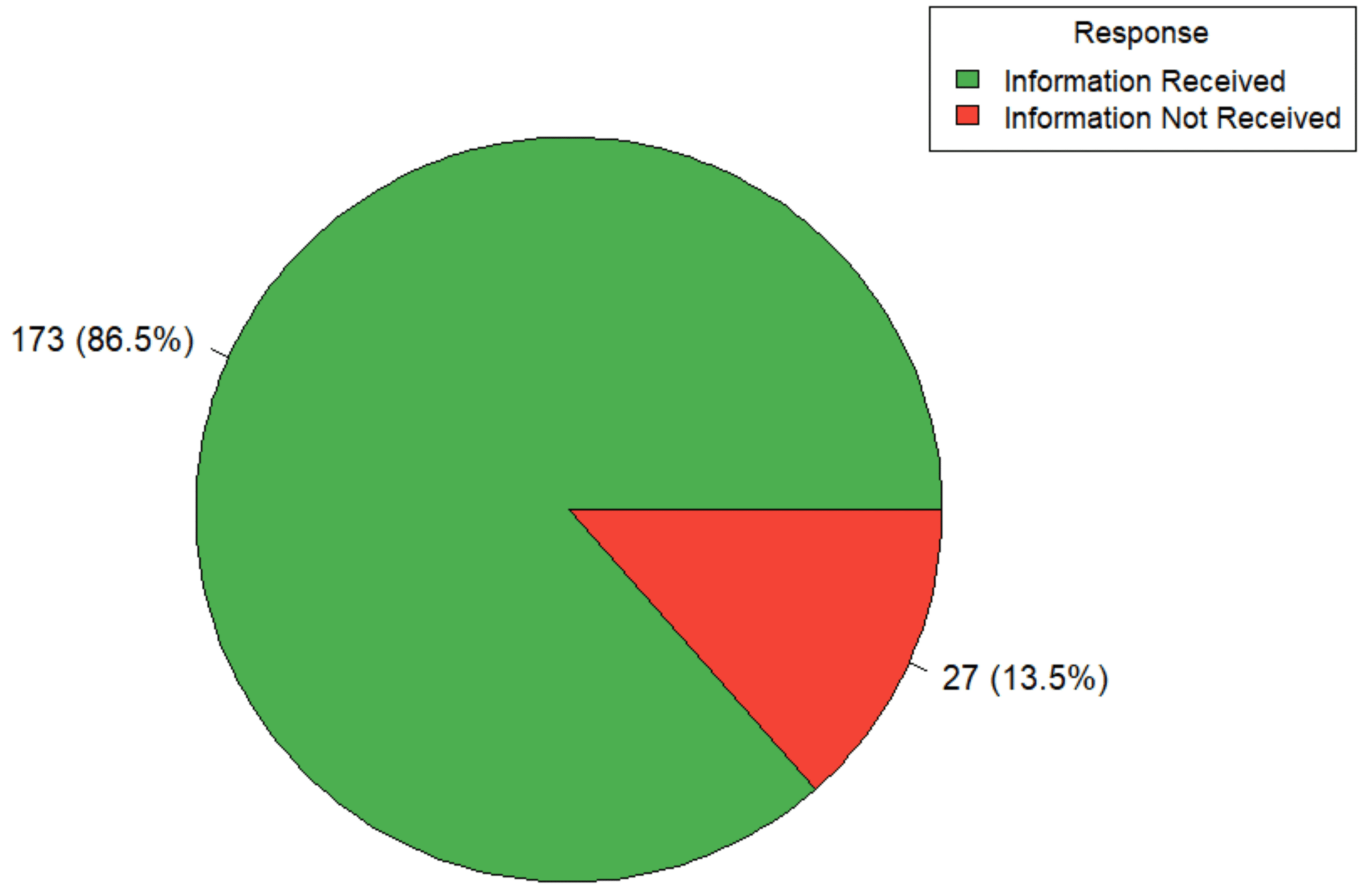
Distribution according to information received regarding anesthesia-related complications (n=200)

All participants (100%, 200/200) reported receiving information about their diagnosis, surgical procedure, anesthesia, and potential surgical complications. However, only 86.5% (173/200) were informed specifically about anesthesia-related complications (Figure 1). This proportion differed significantly based on participants’ place of residence and educational background. Among rural residents, 73.5% (n=61) reported being informed about anesthesia-related complications, compared to 95.7% (n=112) of urban residents. This difference was statistically significant [ꭓ^2 ^(1,200) = 20.55, p-value < 0.001]. Educational level was also significantly associated with being informed about anesthesia-related complications (Fisher’s exact test, p-value < 0.001). Among graduates, 95.3% (n=121) reported receiving this information, while only 4.7% (n=6) did not. In contrast, 52.4% (n=11) of illiterate participants were informed, and 47.6% (n=10) were not. Among those with primary school education, responses were equally divided (50.0%, n=3). Among participants with secondary school education, 82.6% (n=38) were informed and 17.4% (n=8) were not.

**Figure 2:**
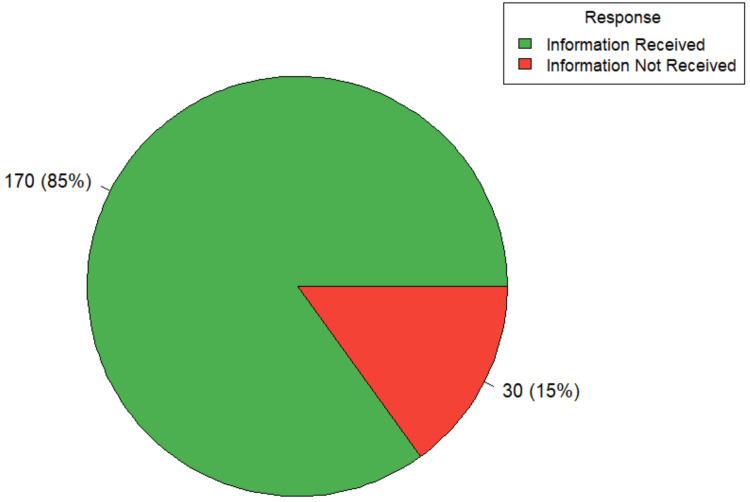
Distribution according to information received regarding alternative treatment options (n=200)

A total of 85.0% (170/200) of participants reported being informed about alternative treatment options (Figure 2). This was significantly associated with both place of residence and the type of surgery performed. Among rural participants, 73.5% (n=61) reported receiving this information, compared to 93.2% (n=109) of urban participants [ꭓ^2^(1,200) = 14.73, p-value < 0.001]. Similarly, 92.4% (n=146) of individuals who had undergone elective procedures were informed about alternative treatments, whereas only 57.1% (n=24) of those who underwent emergency procedures reported the same [ꭓ^2^(1,200) = 32.35, p-value < 0.001]. These findings indicate substantial disparities in the provision of information regarding alternative treatment options.

**Figure 3:**
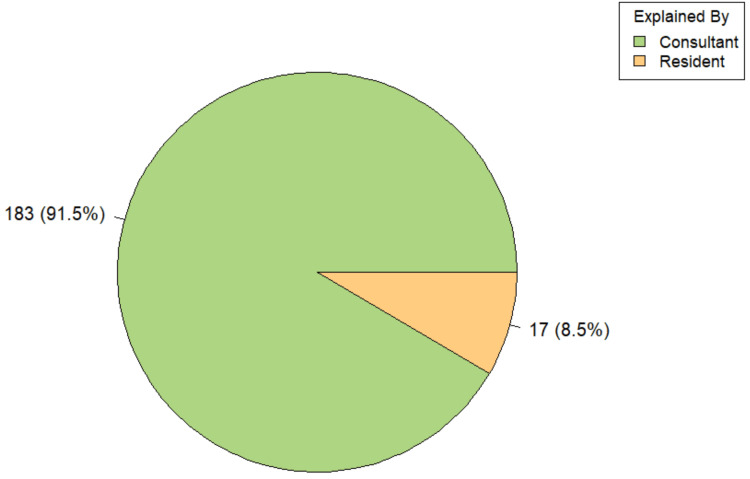
Distribution according to personnel who obtained consent (n=200)

All participants reported being given adequate time to ask questions prior to surgery. In 91.5% (183/200) of cases, consent was obtained by consultants, while in the remaining 8.5% (n=17), it was obtained by residents (Figure 3). The person obtaining consent was significantly associated with both place of residence [ꭓ^2^(1,200) = 6.47, p-value = 0.018] and type of surgery (Fisher’s exact test, p-value < 0.001). Among rural participants, 85.5% (n=71) reported that consent was obtained by a consultant, compared to 95.7% (n=112) among urban participants. Similarly, all individuals who had undergone elective procedures (100%, n=158) reported that consent was taken by a consultant, whereas among those who underwent emergency procedures, only 59.5% (n=25) reported the same. These findings highlight disparities in consent practices, particularly in emergency settings and among rural populations.

**Figure 4:**
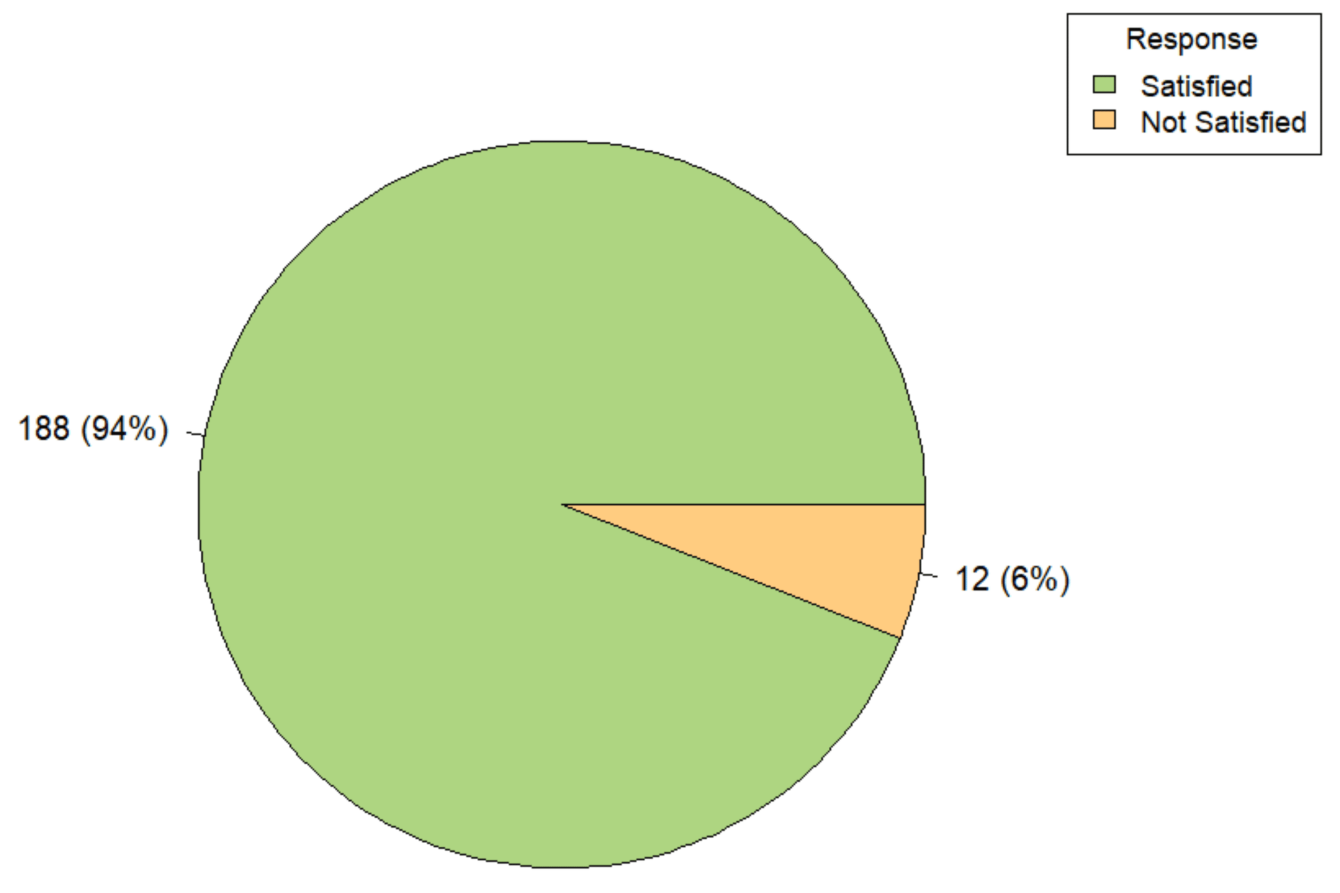
Distribution of satisfaction about consent process among participants (n=200)

In all instances, consent was signed by both patient and attendant, and properly witnessed. However, no audio or video recording was used. The majority of participants (94.0%, 188/200) reported satisfaction with the informed consent process, while only 6.0% (n=12) expressed dissatisfaction (Figure 4). Satisfaction levels varied significantly by place of residence and educational status. Among rural participants, 88.0% (n=73) reported being satisfied, compared to 98.3% (n=115) of urban participants. Dissatisfaction was reported by 12.0% (n=10) of rural and 1.7% (n=2) of urban respondents, with the difference being statistically significant (Fisher’s exact test, p-value=0.003). Satisfaction with the consent process also demonstrated a significant association with educational attainment (Fisher’s exact test, p-value=0.038). Among graduates, 96.1% (n=122) were satisfied, while 3.9% (n=5) were not. In contrast, only 76.2% (n=16) of illiterate participants expressed satisfaction, with 23.8% (n=5) reporting dissatisfaction. All participants with only primary (n=6) and secondary (n=2) education were satisfied, and in the higher secondary school group, 95.5% (n=42) reported satisfaction, with 4.5% (n=2) expressing dissatisfaction.

## Discussion

This study evaluated the quality and comprehensiveness of the preoperative informed consent process among patients who underwent major abdominal surgeries at a tertiary care hospital in Northeast India. The findings indicate high overall satisfaction with the consent process (94%), along with high levels of information provision concerning diagnosis, procedure, and surgical risks. However, gaps remain in the uniformity of information dissemination, especially regarding anesthesia-related risks and alternative treatment options, which were significantly associated with the patients’ educational background, place of residence, and whether the surgery was elective or emergency.

The study revealed that all participants were informed about their diagnosis, surgical procedure, anesthesia, and potential surgical complications, consistent with best practices in the literature that emphasize the importance of comprehensive information disclosure [1]. The overall satisfaction level observed in this study aligns with previous reports. For instance, Patil et al. reported a 96.38% satisfaction rate with the information provided for consent [9]. Falagas et al. also reported relatively high satisfaction among surgical patients but identified significant concerns regarding the depth and clarity of information provided [3]. A study conducted in Ethiopia by Molla et al. revealed that 62% of patients expressed satisfaction, 5% reported being very satisfied, while 33% of patients indicated dissatisfaction with the process [10]. Jawaid et al. documented significantly lower satisfaction with the informed consent process, with only 48.9% of participants expressing satisfaction [11]. Similarly, Anderson and Wearne highlighted that even in well-resourced settings, patients often feel inadequately informed, particularly regarding anesthesia and alternatives to surgery [8]. Our findings reaffirm this global concern, with 13.5% of patients in our study stating they were not informed about anesthesia-related complications.

A notable contribution of this study is the evidence of disparity based on educational level and the urban-rural divide. Graduates were significantly more likely to report receiving comprehensive information than their less-educated counterparts. This echoes findings by Kusec et al., who demonstrated that limited health literacy impairs comprehension of informed consent and emphasized the need for simplified communication tailored to patient literacy levels [2]. Our study’s rural participants also received less comprehensive information than urban patients, consistent with Roberts, who emphasized structural and contextual barriers to effective consent in under-resourced populations [4]. The role of healthcare personnel in obtaining consent also influenced patient perceptions. In our study, when consultants obtained consent, as opposed to residents, patients were more likely to be satisfied, particularly in elective surgeries. This supports findings by Cassileth et al., who suggested that the seniority and communication skills of the healthcare provider play a pivotal role in shaping patient understanding and satisfaction [5].

Despite guidelines mandating documentation [7,12,13], none of the consent processes in this study were audio- or video-recorded. This reflects a broader trend, especially in low-resource settings, where technological integration in ethical documentation remains limited. Future interventions should explore incorporating such tools, especially in emergency contexts where time constraints often compromise the depth of consent discussions.

Interestingly, although all patients acknowledged being informed about their condition and the planned surgical procedure, fewer reported being informed about alternatives (85%) and anesthesia-related risks (86.5%). This discrepancy points to a selective information bias in preoperative communication, wherein life-threatening or immediately relevant aspects are emphasized while subtler but equally important elements may be overlooked. This mirrors patterns observed in prior work by Patil et al., where they reported that 40.72% were informed about alternative surgery [9]. Strikingly, Jawaid et al. reported that only 5% of patients were informed about the potential complications associated with the planned anesthesia [11].

Adhering to the principles of informed consent is crucial, as it involves sharing pertinent details, emphasizing significant risks, and presenting available options, all while ensuring the patient fully understands the information provided. Although this process may appear straightforward, numerous challenges arise in practice, such as language barriers, time and space constraints, varying literacy levels of patients or their relatives, and the use of medical jargon by healthcare professionals [1,14,15].

The present study exhibits several strengths, notably a comprehensive evaluation of the informed consent process, encompassing aspects such as diagnosis, surgical procedures, anesthesia, complications, and alternative treatments. The relatively large sample size of 200 participants enhances the robustness of the findings, and the timing of data collection, between postoperative days 1 and 3, facilitated accurate patient recall. Nonetheless, the study possesses notable limitations. As a single-center study, the generalizability of the results to other settings is restricted. The reliance on self-reported data, without objective measures or documentation review, may introduce bias. Moreover, the absence of a comparison group constrains the ability to contextualize the findings, and there is a lack of comprehensive qualitative exploration regarding dissatisfaction or an assessment of patients' actual comprehension of the information provided.

## Conclusions

This study highlights a generally high level of adherence to informed consent practices for major abdominal surgeries at a tertiary care hospital in Northeast India. Although patient satisfaction with the consent process was substantial, opportunities remain to improve the consistency and depth of information delivery, particularly concerning anesthesia-related risks and alternative treatment options across diverse patient groups. Future research should adopt a multicentric design to improve generalizability and incorporate objective measures, such as documentation audits, to complement self-reported data. Furthermore, qualitative methodologies are warranted to explore underlying reasons for patient dissatisfaction and to evaluate patients’ actual comprehension of the consent information provided.

## Appendices

**Table 2:** Questionnaire used for this study

Assessment of Preoperative Informed Consent Practices Among Patients Who Had Undergone Major Abdominal Surgery: A Cross-Sectional Study in Northeast India						
PATIENT PARTICULARS & DEMOGRAPHY						
S No.			UHID			
Age (in years)						
Gender			Male	Female	Other	-
Residence			Urban	Rural	-	-
Educational Qualification			Illiterate	Primary school	Secondary school	Graduate
Diagnosis						
SURVEY SPECIFIC QUESTIONS *(Please tick the most appropriate one)*						
Q1. Were you informed about your condition before surgery?			Yes	No	-	-
Q2. Were you told the details of the surgery?			Yes	No	-	-
Q3. Were you informed about possible surgical complications?			Yes	No	-	-
Q4. Were you told about the type of anesthesia?			Yes	No	-	-
Q5. Were you informed about anesthesia-related complications?			Yes	No	-	-
Q6. Were alternative treatment options explained to you?			Yes		-	-
Q7. Were you given time to ask questions?			Yes	No	-	-
Q8. Consent was taken by?			Consultant Surgeon	Resident Trainee	Nurse	Others (specify)........................
Q9. Consent was signed/given by?			Patient himself/herself	Family member/attender		
Q10. Was a witness present during the consent process?			Yes	No	-	-
Q11. Were you given enough time to understand the information?			Yes	No	-	-
Q12. Was any audio or video recording done during consent?			Yes	No	-	-
Q13. Are you satisfied with the informed consent process?			Yes	No	-	-
